# Hindering the formation and promoting the dispersion of medical biofilms: non-lethal effects of seagrass extracts

**DOI:** 10.1186/s12906-018-2232-7

**Published:** 2018-05-30

**Authors:** Luca De Vincenti, Yvana Glasenapp, Cristina Cattò, Federica Villa, Francesca Cappitelli, Jutta Papenbrock

**Affiliations:** 1 Dipartimento di Scienze per gli Alimenti, la Nutrizione e l’Ambiente, Università degli Studi di Milano, via Celoria 2, 20133 Milan, Italy; 2 Institute of Botany, Leibniz University Hannover, Herrenhäuserstr. 2, D-30419 Hannover, Germany

**Keywords:** Seagrass extracts, Non-lethal concentrations, Antibiofilm activity, *Escherichia coli*, *Candida albicans*

## Abstract

**Background:**

Biofilms have great significance in healthcare-associated infections owing to their inherent tolerance and resistance to antimicrobial therapies. New approaches to prevent and treat unwanted biofilms are urgently required. To this end, three seagrass species (*Enhalus acoroides*, *Halophila ovalis* and *Halodule pinifolia*) collected in Vietnam and in India were investigated for their effects in mediating non-lethal interactions on sessile bacterial (*Escherichia coli*) and fungal (*Candida albicans*) cultures. The present study was focused on anti-biofilm activities of seagrass extracts, without killing cells.

**Methods:**

Methanolic extracts were characterized, and major compounds were identified by MS/MS analysis. The antibiofilm properties of the seagrass extracts were tested at sub-lethal concentrations by using microtiter plate adhesion assay. The performance of the most promising extract was further investigated in elegant bioreactors to reproduce mature biofilms both at the solid/liquid and the solid/air interfaces. Dispersion and bioluminescent assays were carried out to decipher the mode of action of the bioactive extract.

**Results:**

It was shown that up to 100 ppm of crude extracts did not adversely affect microbial growth, nor do they act as a carbon and energy source for the selected microorganisms. Seagrass extracts appear to be more effective in deterring microbial adhesion on hydrophobic surfaces than on hydrophilic. The results revealed that non-lethal concentrations of *E. acoroides* leaf extract: i) reduce bacterial and fungal coverage by 60.9 and 73.9%, respectively; ii) affect bacterial biofilm maturation and promote dispersion, up to 70%, in fungal biofilm; iii) increase luminescence in *Vibrio harveyi* by 25.8%. The characterization of methanolic extracts showed the unique profile of the *E. acoroides* leaf extract.

**Conclusions:**

*E. acoroides* leaf extract proved to be the most promising extract among those tested. Indeed, the selected non-lethal concentrations of *E. acoroides* leaf extract were found to exert an antibiofilm effect on *C. albicans* and *E. coli* biofilm in the first phase of biofilm genesis, opening up the possibility of developing preventive strategies to hinder the adhesion of microbial cells to surfaces. The leaf extract also affected the dispersion and maturation steps in *C. albicans* and *E. coli* respectively, suggesting an important role in cell signaling processes.

## Background

The ability of microorganisms to colonize surfaces and develop into highly organized communities enclosed in a self-produced polymeric matrix is the predominant growth modality in both nature and artificial systems. Such lifestyle is called biofilm and it is characterized by alterations in microbial phenotypes with respect to growth rates and gene transcriptions [1–3].

Biofilms have great significance for public health, representing 65–80% of microbial diseases currently treated by physicians in the developed world [4, 5]. The presence of indwelling medical devices further increases the risk for biofilm formation and subsequent infection [6]. The bacterium *Escherichia coli* and the polymorphic fungus *Candida albicans* are among the most frequent cause of bloodstream infections, and the predominant microorganisms isolated from infected medical devices [7, 8]. These biofilms, as any other biofilm, exhibit dramatically decreased susceptibility to antimicrobial agents and resistant to the host immune clearance, which increases the difficulties for the clinical treatment of infections [9–11]. Furthermore, the antimicrobial arena is experiencing a shortage of lead compounds, and growing negative consumer perception against synthetic products has led to the search for more natural solutions [12].

In this context, it has been reported that plant-derived extracts exhibit good antibiofilm properties against a range of microorganisms [13–15]. However, in the past, these extracts were mainly screened by focusing on their lethal effects [16–18] disregarding their activity at non-lethal concentrations. At these concentrations, plant-derived extracts may reveal elegant mechanisms to sabotage the sessile lifestyle, manipulating the expression of stage-specific biofilm phenotypes [19]. For instance, by affecting the cellular ability to attach to surfaces and by mystifying intercellular signals, the biofilm cascade might be hampered. Thus, non-lethal concentrations of plant-derived extracts can inspire innovative, eco-friendly and safe strategies aim at treating deleterious biofilms. Interfering with specific key steps that orchestrate biofilm genesis might offer new ways to disarm microorganisms without killing them, sidestepping drug resistance [4].

Seagrasses, which belong to the halophytes, represent a functional group of underwater marine flowering plants that have developed several strategies to survive and reproduce in environments where the salt concentration is around 200 mM NaCl or more [20]. As these plants grow in very high saline conditions, it is predicted that they could possess rare and new activities not reported for their terrestrial relatives [21, 22]. Indeed, metabolomic studies have shown that increased salinity leads to changes in conserved and divergent metabolic responses in halophytes [23–25]. Moreover, interesting activities of seagrass extracts, including antibacterial, antifungal, antialgal, antioxidant, anti-inflammatory, insecticidal, antimalarial and vasoprotective properties, have been reported [26–28].

Thus, the well described properties of seagrasses extracts offer a promising framework for investigating novel antibiofilm activities at non-lethal concentrations.

The present study explores, for the first time, the effect of extracts from different seagrasses (namely, leaves and roots from *Enhalus acoroides* Rich. ex Steud., Hydrocharitaceae, leaves of *Halophila ovalis* (R.Br.) Hook.f., Hydrocharitaceae, and leaves of *Halodule pinifolia* (Miki) Hartog*,* Cymodaceaceae) in mediating non-lethal interactions on sessile *Candida albicans* and *Escherichia coli* cultures, selected as model systems for fungal and bacterial biofilm infections, respectively. The work focuses on investigating the antibiofilm performance of seagrass extracts at sub-inhibitory concentrations, studying how they affect biofilm functional traits (such as adhesion, biofilm maturation, dispersal and quorum sensing), and induce cellular responses other than those associated with antimicrobial activities.

## Methods

### Plant material and extraction

Three species of seagrasses (leaves and roots from *Enhalus acoroides* Rich. ex Steud., Hydrocharitaceae, leaves of *Halophila ovalis* (R.Br.) Hook.f., Hydrocharitaceae, and leaves of *Halodule pinifolia* (Miki) Hartog*,* Cymodaceaceae) were collected in Vietnam and India and air-dried in a dark place (Table 1). *Enhalus acoroides* and *Halophila ovalis* were collected and identified by Xuan-Vy Nguyen, Department of Marine Botany, Institute of Oceanography, Vietnam Academy of Science and Technology, Nha Trang City, Vietnam, based on morphological characters and controlled by ITS molecular marker analysis [29]. Specimens of *Enhalus acoroides* are stored in the herbarium of the Institute of Botany, Hannover, Germany (Specimen number: EA20130301). *Halodule pinifolia* was collected by Jutta Papenbrock and further identified by Thirunavakkarasu Thangaradjou, Centre of Advanced Study in Marine Biology, Annamalai University, Parangipettai, Tamilnadu, India, based on morphological characters and controlled by ITS molecular marker analysis [30]. Specimens are stored in the herbarium of the Annamalai University, Parangipettai, Tamilnadu, India.

**Table 1:** Seagrass species and information about collection sites

Species	Plant organ	Collection site	GPS	Collection date
*Enhalus acoroides*	Leaf	Nha Trang Bay, Vietnam	109.209208°E 12.158073°N	19.04.2011
*Enhalus acoroides*	Root	Nha Trang Bay, Vietnam	109.209208°E 12.158073°N	19.04.2011
*Halophila ovalis*	Leaf	Nha Trang Bay, Vietnam	109.209208°E 12.158073°N	19.04.2011
*Halodule pinifolia*	Leaf	Chilika Lagoon, India	85.418015°E 19.775105°N	16.02.2010

The plants were separated into different organs (leaves and roots), and samples were cooled with liquid nitrogen and ground to a fine powder using a bead mill (Retsch), three times for 10 s at a frequency of 30/s. The samples were stored at − 80 °C prior to analysis. Crude extracts were obtained using 80% methanol (MeOH) as solvent. Around 50 mg of powdered seagrass material was weighed in a reaction tube and extracted with 800 μl 80% MeOH for 10 min with regular shaking. Then the extract was centrifuged for 5 min at 18000 x g and the supernatant transferred into a new reaction tube. These steps were repeated three times with 400 μl 80% MeOH each. The supernatants were collected in the same reaction tube and stored at − 20 °C. Phosphate buffered saline (PBS, 0.01 M phosphate buffer, 0.0027 M potassium chloride 0.137 M, sodium chloride, Fisher Scientific) was used to obtain several concentrations of each crude extract: 100, 10, 1, 0.1, 0.01 and 0.001 mg/l.

### Microbial strains and growth media

The microbial strains *Candida albicans* SC5314 (ATCC MYA-2876) and *Escherichia coli* K-12 wild-type strain (ATCC 25404) were selected as model systems for fungal and bacterial biofilms respectively. *C. albicans* and *E. coli* strains were stored at − 80 °C in suspensions containing 50% glycerol and 2% peptone, and were routinely grown in amino acid-free yeast nitrogen base (YNB, Sigma-Aldrich) supplemented with 0.5% glucose (YNBG, Conda) and Luria-Bertani broth (LB, Sigma-Aldrich), respectively, for 16 h at 30 °C.

### Quantification of total flavonoid contents (TFC)

The total flavonoid content of the seagrass extracts was measured in 96-well plate according to a modified protocol from Dudonné et al. [31]. The wells were filled with 150 μl H_2_O each. Dilutions of the methanolic seagrass extracts (1:2) were prepared and 25 μl of sample were filled in one well, with four replicates. A calibration curve with catechin hydrate with the following concentrations was prepared in 80% MeOH: 0, 10, 25, 50, 100, 125, 250 and 400 μg/ml. The calibration curve was placed on the plate in triplicate. In the next step, 10 μl NaNO_2_ 3.75% were added into each well and incubated for 6 min. Afterwards, 15 μl of AlCl_3_ 10% were added and incubated for 10 min. In the last step, 50 μl of NaOH 1 M were added and the absorption was measured at 510 nm in a microplate reader (Biotek, Winooski, USA). The slope of the calibration curve was used to calculate the total flavonoid content in mg catechin equivalent.

### Quantification of total phenolic contents (TPC)

To measure the total phenolic acid content, a modified protocol after Dewanto et al. [32] was used with the same extracts described above. 96-well microtiter plate were filled with 100 μl H_2_O each. From each sample, 10 μl were added; seagrass extracts were diluted 1:2. A gallic acid calibration curve with the following concentration was used: 0, 5, 10, 25, 50, 75, 100, 125 and 250 μg/ml. Next, 100 μl Na_2_CO_3_ 7% were added and the plate was incubated for 100 min in the dark. The absorption was measured at 765 nm in a microplate reader. With the slope of the gallic acid calibration curve, the concentration of phenolic acids was calculated in mg gallic acid equivalent.

### Determination if the oxygen radical absorbance capacity (ORAC)

The analysis of the oxygen radical absorbance capacity (ORAC) was conducted according to a protocol based on Huang et al. (2002) [33] and Gillespie et al. [34] with the same extracts. A black 96-well microtiter was used and the wells were filled with 120 μl fluorescein (112 nM) in phosphate buffer (75 mM, pH 7.4). Of each sample and the standard curve, 20 μl were added in each well. The standard curve of 6-hydroxy-2,5,7,8-tetramethylchroman-2-carboxylic acid (Trolox) was prepared in phosphate buffer with the following concentrations: 6.25, 12.5, 25 and 50 μM. Seagrass extracts were diluted 1:200 with methanol 80%. The microtiter plate was incubated for 15 min at 37 °C. The fluorescence was then measured at 485/520 nm as time point zero. Next, 80 μl of 2,2′-azobis(2-amidino-propane) dihydrochloride (62 mM) were added and the fluorescence was measured every minute for 80 min. The ORAC value was calculated as the difference between time point zero and 80 min and quantified with the Trolox standard curve.

### LC-MS analysis

LC-MS analysis was performed on a Shimadzu HPLC system (controller CBM-20A, two pumps LC-20 AD, a column oven CTO-20 AC and a photo diode array detector SPD-M20A; Shimadzu, Darmstadt, Germany) coupled to a Triple Tof 4600 mass spectrometer (AB Sciex, Canby, USA). The separation of extracted compounds was realised on a Knauer Vertex Plus column (250 × 4 mm, 5 μm particle size, packing material ProntoSIL 120–5 C18-H) with precolumn (Knauer, Berlin, Germany). The column oven temperature was set to 30 °C and 25 μl of undiluted methanolic seagrass extract prepared as described above was injected. The solvent flow rate was 0.8 ml/min. In this time, a gradient was run from 10 to 90% B from minute 0 to 35, 2 min of 90% B, switch to 10% B in 1 min and subsequent equilibration at 10% B for 2 min. Solvent A (water) and B (methanol) were both supplemented with 2 mM ammonium acetate and 0.01% acetic acid. Mass spectra were monitored between 100 and 800 Da in negative ionisation mode. In addition, MS/MS spectra were generated with a collision energy of − 30 eV and measured between 50 and 800 Da. Spectra for the most prominent peaks were compared to database entries in MassBank [35] and ReSpect [36] for identification.

### Planktonic growth in the presence of seagrass extracts as the sole source of carbon and energy

The ability of *C. albicans* and *E. coli* planktonic cells to grow in the presence of each extract as the sole carbon and energy source was tested using YNB and M9 (Sigma-Aldrich) mineral medium, respectively, supplemented with the highest working extract concentration: 100 mg/l. Then a 100 μl mix of mineral medium together with 45 μl (3% *v*/v) of the overnight culture (final concentration 10^8^ cells/ml) and the highest concentration of each marine plant extract were used to fill each well of 96-well plates (Thermo Fisher Scientific) and incubated for 48 h at 30 °C. A medium complemented with cells and glucose (5 g/l), and medium without cells, were used as positive and negative controls, respectively. Microbial growth was monitored using the PowerWave XS2 microplate reader (Biotek) measuring the absorbance at 600 nm (A_600_) every 10 min. Six biological replicates of each treatment were performed. The obtained data were normalized to the negative control and reported as the mean of these.

### Growth inhibition assay in the presence of seagrass extracts

The ability of the seagrass extracts to inhibit the planktonic growth of the selected microorganisms was investigated. For this, *C. albicans* and *E. coli* were grown YNBG and LB broth respectively without (positive control) and with the highest working concentrations (10 and 100 mg/l) in 96-well plates (Thermo Fisher Scientific). Growth curves at 30 °C were generated using Infinite® F200 PRO microplate reader (TECAN, Mannedorf, Switzerland) by measuring the optical density at 600 nm (OD_600_) every 60 min for 30 h in wells inoculated with 45 μl (3% vol/vol) of an overnight culture (approximately 10^8^ cells/ml). The negative control was represented by PBS supplemented with 45 μl (3% vol/vol) of the overnight culture. The polynomial Gompertz model [37] was used to fit the growth curves to calculate the maximum specific growth rate (A_600_/min), using GraphPad Prism software (version 5.0, San Diego, CA, USA). Five biological replicates of each treatment were performed.

### Microplate-based biofilm assay

The antibiofilm activity of seagrass extracts was assessed quantitatively as previously reported by Villa et al. [38]. Briefly, 200 μl of PBS containing 10^8^ cells/ml supplemented with 0 (positive control), 100, 10, 1, 0.1, 0.01, and 0.001 mg/l of each crude extract were placed in hydrophobic and hydrophilic 96-well polystyrene-based microtiter plates (Thermo Fisher Scientific). After an incubation time of 24 h at 20 °C, *C. albicans* and *E. coli* planktonic cells were removed and adhered cells were stained using 0.1 mg/ml of Fluorescent Brightener 28 vital dye (Sigma-Aldrich) or 4′, 6-diamidino-2-phenylindole (DAPI, Sigma-Aldrich) in PBS, respectively. After 20 min staining in the dark at room temperature the microtiter plates were washed twice with 200 μl PBS and the fluorescence intensity due to adhered cells was measured using a fluorescence microplate reader (TECAN, Manneford, Switzerland) at excitation wavelength of 335 nm and emission wavelength of 433 nm. A standard curve of fluorescence intensity versus cell number was determined and used to quantify the antibiofilm performance of the crude extracts. Percentage reduction with respect to the positive control is calculated as (treated data –control data) × 100 / control data. Cattò et al. [39] proposed the following anti-adhesion ranges computing the percentage reduction in comparison to the negative control: ≤20% without anti-adhesion activity; between 20 and 30% and 30 and 40% low anti-adhesion activity and with moderate anti-adhesion activity respectively; ≥40% adhered cells with excellent anti-adhesion activity. Five biological replicates were performed for each condition and a percentage reduction in comparison to the negative control was calculated as (treated data – positive control data) × 100/positive control data. The experiment was repeated three times.

### Biofilm growth at the solid/liquid interface

The most promising plant extracts were screened for their effects on biofilm development. *C. albicans* biofilm was grown in the CDC biofilm reactor (Biosurface Technologies, Bozeman, MT, USA) as previously described by Villa et al. [40]. Briefly, two bioreactors hosting 24 polycarbonate coupons (to simulate a hydrophobic surface) were filled with YNBG and 1 ml of overnight planktonic culture (approximately 10^8^ cells/ml) and, in one of them, 0.01 mg/l of *E. acoroides* leaf extract was added. Bioreactors were maintained under static conditions (no flow) for 24 h under mild stirring at 37 °C, promoting fungal adhesion to the surface of the removable polycarbonate coupons. After that, the dynamic phase was initiated and diluted YNGB was fluxed for 48 h at flow rate of 250 ml/h. Biofilm growth in the absence (positive control) and presence of the extract was evaluated by quantification of the biomass. At different time steps (24, 48 and 72 h) some polycarbonate coupons were collected in aseptic conditions and resuspended in 3 ml of PBS each. Subsequently, serial dilutions were carried out, and 10 μl were inoculated in petri dishes containing Tryptic Soy Broth medium (TSB, Sigma-Aldrich) complemented with agar (Merck) following the drop counting method. After 12 h at 30 °C, *C. albicans* colonies were counted and the data obtained were normalized to the coupon area, and means were reported. The same protocol was used to obtain mature biofilm of *E. coli,* using LB as a medium, and evaluating 10 mg/l of *E. acoroides* leaf extract. Each experiment was repeated three times.

### Biofilm dispersion assay

Mature *C. albicans* biofilm was grown in the CDC reactor in the absence (positive control) and presence and of 0.01 mg/l of *E. acoroides* leaf extract as reported below. As previously described by Cattò et al. [41], after 72 h polycarbonate coupons were collected, immersed in 27 ml of PBS for one minute at room temperature, serial dilutions were carried out and 10 μl were inoculated in petri dishes containing TSB supplemented with agar (Merck) following the drop counting method. After 12 h at 30 °C, *C. albicans* colonies were counted and the percentage of biofilm dispersion was calculated as (number of viable cells from bulk PBS × 100) / (number of viable cells from bulk PBS + number of viable cells from the coupon biofilm) and means were reported. Three biological replicates were performed for each treatment and six technical replicates were performed for each experiment. The experiment was performed three times.

### Biofilm growth at the solid/air interface

*E. coli* biofilm was grown on a sterile polycarbonate membrane (PC, Whatman Nucleopore, diameter 2.5 cm, pore diameter 0.2 μm) as previously described by Garuglieri et al. [42]. Briefly, 0.05 ml of an overnight culture (approximately 10^6^ cells/ml) were inoculated at the center of a sterile polycarbonate membrane and, when the inoculum was completely dried, the membrane was carefully put inside a transwell structure (ThinCert™ Cell Culture Inserts with translucent PET membrane – Greiner bio-one) inlaid in a 6 well culture plate (Greiner bio-one). One ml of LB medium was inoculated in the basolateral compartment (plate well). Biofilm formation was performed at 37 °C in aerobic conditions for 16 h. At different time points (0, 4, 6, 8, 16 h) some membranes were removed, biofilm was scraped off using a sterile loop, put inside a tube containing 1 ml of PBS and then homogenized twice using a homogenizer (IKA T10 basic Ultra-Turrax – Cole-Parmer Instrument Company). Then serial dilutions were prepared and 10 μl were inoculated in petri dishes containing LB with agar following the drop counting method. After 12 h at 37 °C, *E. coli* colonies were counted and the biomass was quantified. This assay was assessed under three experimental conditions: i) treatment 1: growth in contact with 1 ml of LB with 10 mg/l of *E. acoroides* leaf extract for 16 h; ii) treatment 2: overnight culture grown with 10 mg/l of *E. acoroides* leaf extract, and then growth in contact with 1 ml of LB for 16 h; iii) treatment 3: overnight culture grown with 10 mg/l of *E. acoroides* leaf extract, and then growth in contact with 1 ml of LB with 10 mg/l of *E. acoroides* leaf extract for 16 h. In the positive control, the microorganisms grew in 1 ml LB inside a basolateral well for 16 h without the extract. The data obtained were divided by the area of the membrane, and the means were reported. The experiment was repeated three times.

### B2ioluminescence assay using *Vibrio harveyi*

Two hundred μl of autoinducer bioassay (AB) mineral medium (0.3 M NaCl, 0.05 M MgSO_4_, 0.5% casein hydrolysate, 10 μM KH_2_PO_4_, 1 μM L-arginine, 50% glycerol, 0.01 μg/ml riboflavin, 1 μg/ml thiamine. pH 7. Sigma-Aldrich) containing 10% (*V*/V) of a tenfold dilution of an overnight culture of *Vibrio harveyi* BB170 (ATCC BAA-1117) grown in AB medium were supplemented with 10 mg/l of *E. acoroides* leaf extract respectively, and were placed in hydrophobic 96-well polystyrene-based microtiter plates (Thermo Fisher Scientific) with transparent bottom. The positive control was an AB mineral medium supplemented with 10% (V/V) tenfold dilution of the overnight culture. Absorbance (OD_600nm_) and luminescence were measured using a microplate reader (VICTOR™X, Perkin Elmer, USA) every 8 h for 24 h, incubating the microtiter plate at 30 °C during the experiment. The data obtained were normalized to the number of viable cells, divided by the area of the membrane, and the means reported. The experiment was repeated three times.

### Statistical analysis

To evaluate statistically significant differences among samples, analysis of variance (ANOVA) via MATLAB software (Version 7.0, The MathWorks Inc., Natick, USA) was applied. Tukey’s honestly significant different test (HSD) was applied for pairwise comparison to establish the significance of the data. Statistically significant results were represented by *P* values ≤0.05.

## Results

### Seagrass extracts contain phenolic compounds and show antioxidant capacities

**Fig. 1:**
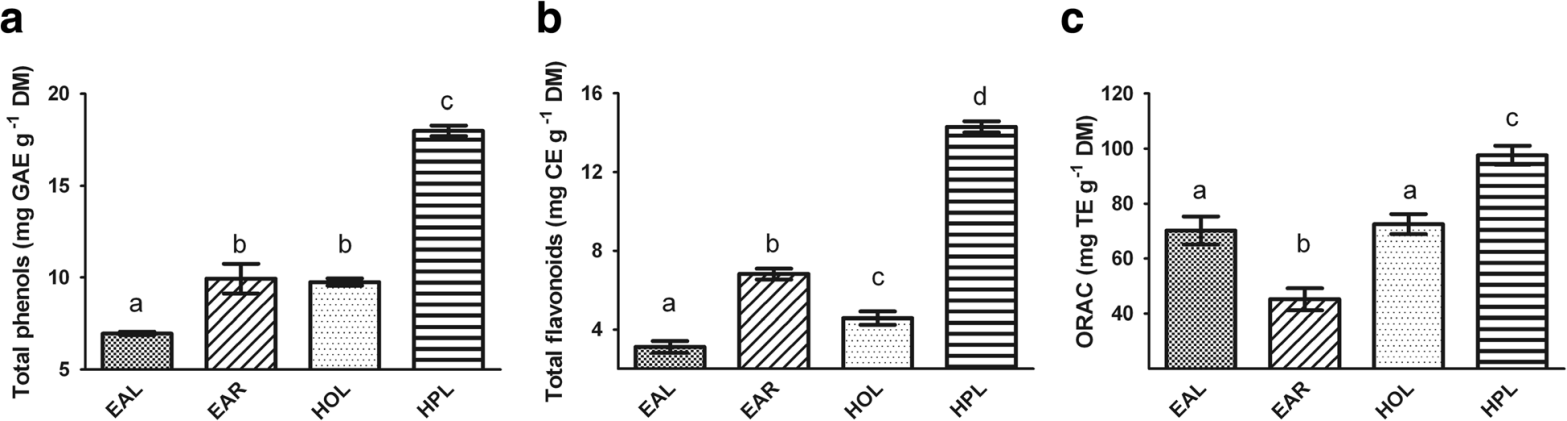
Crude methanolic extracts were analyzed for (**a**) Total phenols in mg gallic acid equivalent (GAE) per g dry mass (DM), (**b**) Total flavonoids in mg catechin equivalent (CE) per g DM, and (**c**) ORAC in mg Trolox equivalents (TE) per g DM. Data represent the mean ± SDs and different superscript letters indicate statistically significant differences (Tukey’s HSD, *p* ≤ 0.05) between the means of three independent measurements. (EAL = *Enhalus acoroides* leaf; EAR = *Enhalus acoroides* root; HPL = *Halodule pinifolia* leaf; HOL = *Halophila ovalis* leaf)

The methanolic extracts from the seagrass material contained phenolic acids as well as flavonoids (Fig. 1a-b). The content of phenols and flavonoids was highest in *H. pinifolia* leaf extracts with 18.0 ± 0.25 and 14.3 ± 0.25 mg/g dry mass (DM), respectively. In *E. acoroides*, the root material showed higher amounts of total flavonoids and phenols than the leaf material. For all seagrass species, the content of phenolic acids was higher than the flavonoid content with respect to the DM.

Methanolic extracts from the four seagrass species were analyzed for their antioxidant capacity (Fig. 1c). All tested extracts had the ability to absorb oxygen radicals. *H. pinifolia* showed the highest activity with 97.7 ± 2.7 mg Trolox equivalents (TE)/g DM. *E. acoroides* and *H. ovalis* leaf extracts showed similar antioxidant capacities with 70.2 ± 4.1 and 72.5 ± 2.9 mg TE/g DM, respectively. The root extract from *E. acoroides* displayed a lower ORAC value than the extract from the leaves (45.1 ± 3.2 mg TE/g DM).

### LC-MS analysis of secondary metabolites

**Fig. 2:**
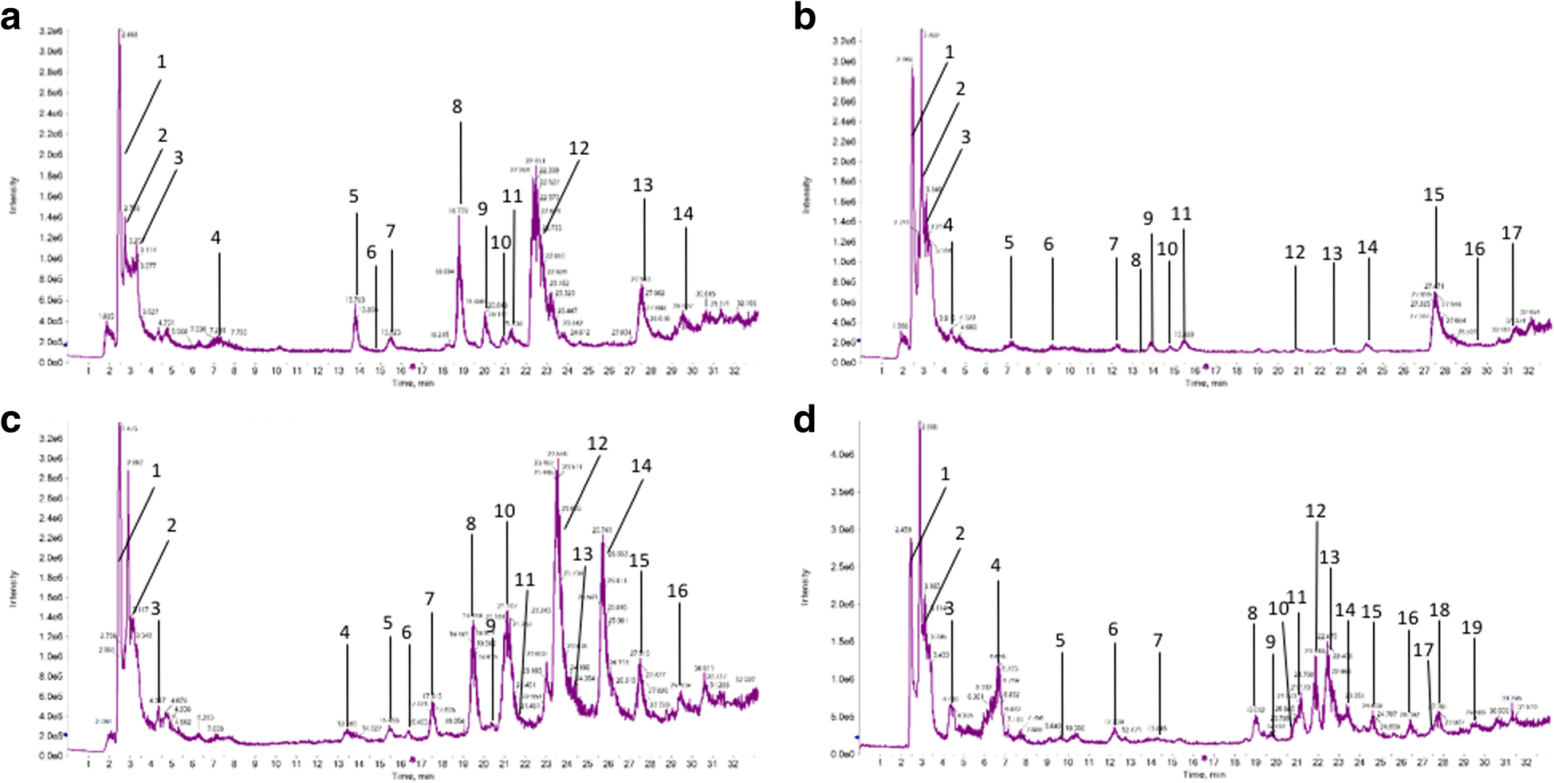
Chromatograms from *E. acoroides* leaf extract (**a**), *E. acoroides* root extract (**b**), *H. ovalis* leaf extract (**c**) and *H. pinifolia* leaf extract (**d**) from minute 0–33. The relative intensity of mass between 100 and 800 Da is shown. Numbers indicate putatively identified substances in Table 2

*E. acoroides*, *H. ovalis* and *H. pinifolia* show different compositions of secondary metabolites (Fig. 2). The identification of individual compounds in the methanolic extracts was done via the comparison of MS/MS spectra with database entries. The three seagrass species showed different profiles of secondary metabolites, in this case mainly flavonoids and phenolic acids (Table 2). In *E. acoroides* leaves, three flavonoles based on kaempferol were found. In addition, two flavones (apigenin and luteolin), one phenolic acid (benzoic acid) and the saturated dicarboxylic acid azelaic acid were identified. The root extract of *E. acoroides* also contained two kaempferol-based flavonoles and luteolin and also a procyanidin and a flavanole (epicatechin). In *H. ovalis* three flavonoids and one phenolic acid was found. *H. pinifolia* contained several flavonoles, either based on kaempferol or quercetin and also epicatechin.

**Table 2:** Individual compounds identified by comparison of MS/MS spectra with database entries in *Enhalus acoroides* leaf extract (A), *E. acoroides* root extract (B), *Halophila ovalis* leaf extract (C) and *Halodule pinifolia* leaf extract (D)

No	RT	Mass	MS/MS	Name	Accession	Source
A - *E. acoroides* leaf extract						
1	2.5	343.03	201.02, 157.03, 59.01	n. i.	–	–
2	2.7	312.12	179.05, 132.06, 89.02	n. i.	–	–
3	3.3	367.1	277.07, 187.04, 157.03	n. i.	**–**	**–**
4	7.2	134.04	107.03, 92.02	Adenine	PT200393	ReSpect
5	13.7	637.1	461.07, 285.04	Kaempferol-3-glucuronide, mod.	PT209240	ReSpect
6	14.8	275.15	233.12, 119.05	n. i.	–	–
7	15.2	121.03	92.02, 77.03	Benzoic acid	KO000321	MassBank
8	18.6	527.02	285.04, 241.00, 96.96	n. i.	**–**	**–**
9	20.1	511.05	269.04, 241.00, 96.96	n. i.	**–**	**–**
10	20.8	187.09	169.08, 125.09, 97.06	Azelaic acid	KO000124	MassBank
11	21.3	447.09	285.04	Kaempferol-3-O-glucoside	PS042209	ReSpect
12	22.5	461.07	285.04	Kaempferol-3-glucuronide	PS092408	ReSpect
13	27.5	285.04	151.00, 133.03	Luteolin	PS040410	ReSpect
14	29.5	269.04	225.05, 151.00, 117.03	Apigenin	PT203930	ReSpect
B - *E. acoroides* root extract						
1	2.4	343.03	201.02, 157.03, 59.01	n. i.	–	–
2	2.7	312.12	179.05, 132.06, 89.02	n. i.	–	–
3	2.9	377.08	341.11, 179.05, 119.03, 89.02	Galactinol dihydrate, mod.	PT211910	ReSpect
4	4.3	216.98	173.02, 156.98, 136.94, 59.01	n. i.	–	–
5	7.2	134.04	107.03, 92.02	Adenine	PT200393	ReSpect
6	9.6	577.12	451.10, 425.08, 407.07, 289.07, 125.02	Procyanidin B2	PT204580	ReSpect
7	12.3	289.07	245.07, 203.07, 151.04, 109.03	+(−) Epicatechin	PT204560	ReSpect
8	13.8	637.1	461.07, 285.04	Kaempferol-3-glucuronide, mod.	PT209240	ReSpect
9	14.0	469.08	275.02, 193.05, 178.02, 149.06, 96.96	n. i.	–	–
10	14.8	275.15	233.12, 119.05	n. i.	–	–
11	15.3	121.03	92.02, 77.03	Benzoic acid	KO000321	MassBank
12	20.8	187.09	169.08, 125.09, 97.06	Azelaic acid	KO000124	MassBank
13	22.6	461.07	285.04	Kaempferol-3-glucuronide	PS092408	ReSpect
14	24.1	299.05	284.03, 256.03, 133.03	Kaempferide	PT204030	ReSpect
15	27.5	285.04	151.00, 133.03	Luteolin	PS040410	ReSpect
16	29.5	269.04	225.05, 151.00, 117.03	Apigenin	PT203930	ReSpect
17	31.2	329.23	229.14, 211.13, 171.10	n. i.	–	–
C - *H. ovalis* leaf extract						
1	2.4	343.03	201.02, 157.03, 59.01	n. i.	–	–
2	2.9	377.08	341.11, 179.05, 119.03, 89.02	Galactinol dihydrate, mod.	PT211910	ReSpect
3	4.3	216.98	173.02, 156.98, 136.94, 59.01	n. i.	–	–
4	13.3	261.04	217.05, 189.05, 133.02	n. i.	–	–
5	15.5	121.03	92.02, 77.03	Benzoic acid	KO000321	MassBank
6	16.3	306.17	288.16	n. i.	–	–
7	17.5	479.08	316,02	Myricetin-3-galactoside	PS092809	ReSpect
8	19.5	463.09	301,03	Quercetin-3-O-beta-D-galactoside	PS046509	ReSpect
9	20.8	187.09	169.08, 125.09, 97.06	Azelaic acid	KO000124	MassBank
10	21.1	317.02	271.02, 149.02	n.i.	–	–
11	21.3	447.09	285.04	Kaempferol-3-O-glucoside	PS042209	ReSpect
12	23.5	301.03	255.03, 165.02, 133.03	n.i.	–	–
13	24.1	299.05	284.03, 256.03, 133.03	Kaempferide	PS040309	ReSpect
14	25.7	285.04	239.03, 185.06, 143.05, 117.03	Kaempferol	PR040027	MassBank
15	27.5	285.04	285.04, 151.00,133.02	Luteolin	PT204043	ReSpect
16	29.4	269.04	225.05, 151.00, 117.03	Apigenin	PT203930	ReSpect
D - *H. pinifolia* leaf extract						
1	2.4	343.03	201.02, 157.03, 59.01	n. i.	–	–
2	2.9	377.08	341.11, 179.05, 119.03, 89.02	Galactinol dihydrate, mod.	PT211910	ReSpect
3	4.3	216.98	173.02, 156.98, 136.94, 93.03, 59.01	n. i.	–	–
4	6.6	473.07	311.04, 293.03, 179.03, 149.01	n. i.	–	–
5	9.6	577.12	451.10, 425.08, 407.07, 289.07, 125.02	Procyanidin B2	PT204580	ReSpect
6	12.1	289.07	245.07, 203.07, 151.04, 109.03	+(−) Epicatechin	PT204560	ReSpect
7	14.0	469.08	275.02, 193.05, 178.02, 149.06, 96.96	n. i.	–	–
8	19.1	641.17	473.13, 311.07, 167.03	n. i.	–	–
9	19.7	549.09	505.10, 463.09, 300.02, 271.02, 255.02	Quercetin-3-(6-malonyl)-glucoside	PT209340	ReSpect
10	20.8	187.09	169.08, 125.09, 97.06	Azelaic acid	KO000124	MassBank
11	21.1	505.09	463.08, 300.02, 271.02	Quercetin-3-O-beta-D-galactoside, mod.	PT204650	ReSpect
12	21.8	463.08	300.03, 271.02	Quercetin-3-O-beta-D-galactoside	PT204650	ReSpect
13	22.4	433.07	300.02, 271.02, 255.03, 179.00	Quercetin-3-arabinoside	PT209320	ReSpect
14	23.4	447.09	284.03, 255.03, 227.03	Kaempferol-3-glucoside	PT209270	ReSpect
15	24.6	417.08	284.03, 255.03, 227.03	Kaempferol-3-O-alpha-L-arabinoside	PT209220	ReSpect
16	26.3	301.03	178.99, 151.00, 121.03, 107.01	Quercetin	PT204090	ReSpect
17	27.4	285.04	199.03, 175.04, 151.00, 133.02	Luteolin	PT204043	ReSpect
18	27.7	315.05	300.02, 271.02, 255.03	Isorhamnetin	PM007432	ReSpect
19	29.5	269.04	225.05, 151.00, 117.03	Apigenin	PT203930	ReSpect

No = number of peak in Fig. 9, RT = retention time, Mass = mass of precursor ion, MS/MS = fragment spectra obtained at − 30 eV, Accession = accession number in database, Source = database used, n. i. = not identified, mod. = modified

### Seagrass extracts are not used as carbon and energy source by *C. albicans* and *E. coli* and do not affect their planktonic growth

**Fig. 3:**
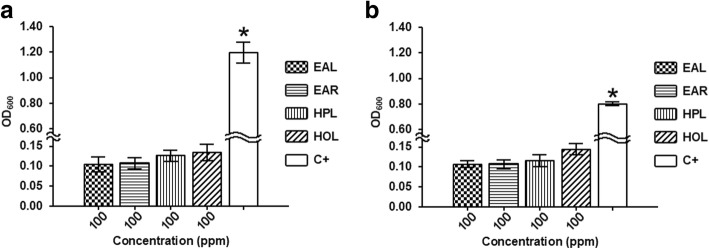
*E. coli* (**a**) and *C. albicans* (**b**) planktonic growth without (positive control) and with each seagrass extract at 100 ppm. The positive control was set up with mineral medium supplemented with glucose at 5 g/l. Stars indicate statistically significant differences (Tukey’s HSD, *p* ≤ 0.05) between the means of three independent replicates. (EAL = *Enhalus acoroides* leaf; EAR = *Enhalus acoroides* root; HPL = *Halodule pinifolia* leaf; HOL = *Halophila ovalis* leaf; C + = Positive control)

*C. albicans* and *E. coli* planktonic cells grown only in the presence of medium supplemented with glucose were used as the positive control of the experiment (Fig. 3). Note that the mineral medium supplemented with the highest concentration of tested plant extracts did not promote the growth of the selected microorganisms.

**Fig. 4:**
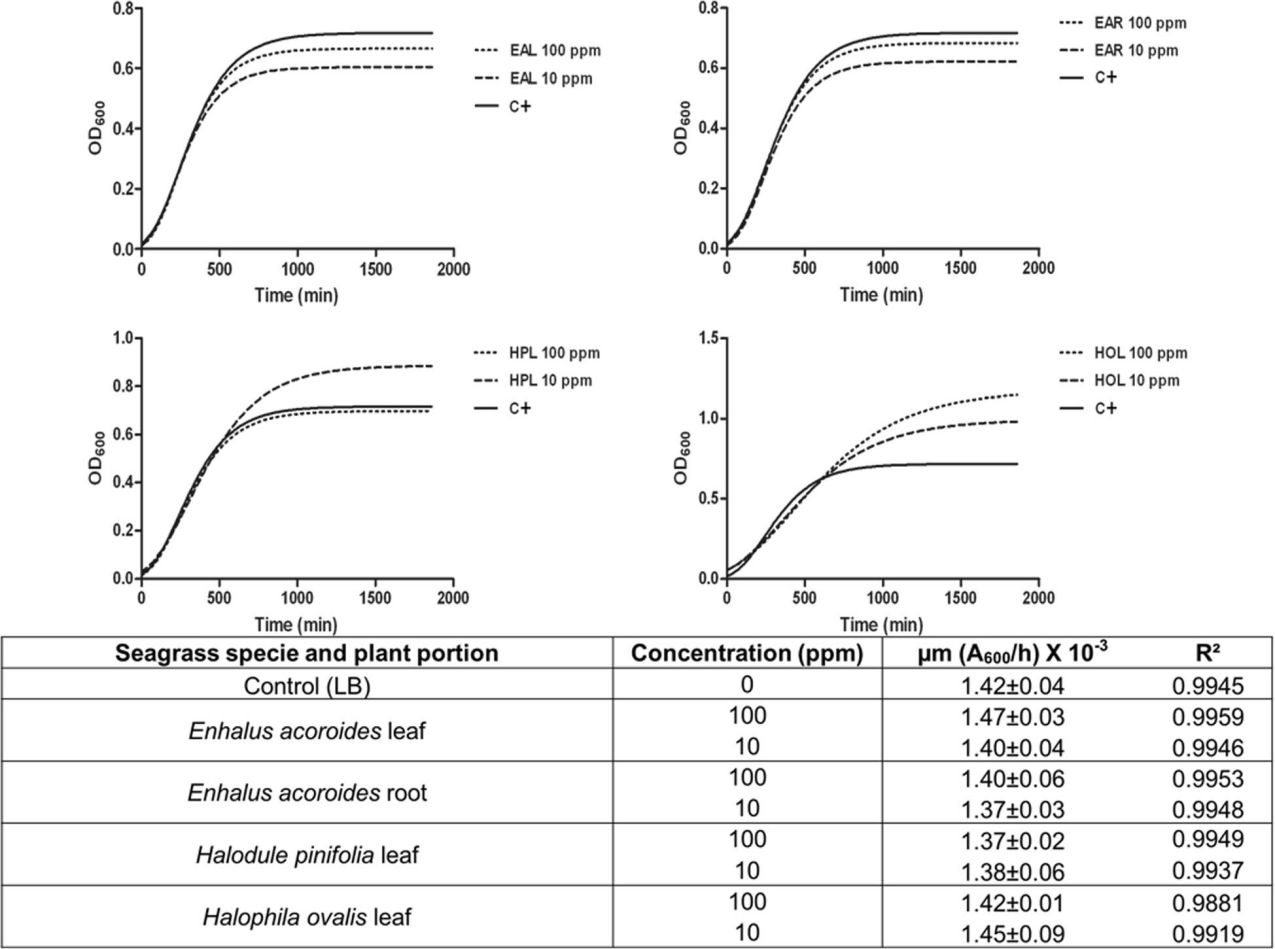
OD-based growth curves of *C. albicans* in absence (positive control) and in presence of each seagrass extract at 10 and 100 ppm. Maximum specific growth rate (μ_m_) and the goodness of fit (R^2^) obtained by the Gompertz model. Data represent the mean ± SDs of three independent measurements. Means reported showed no statistically significant differences between the positive control and treated samples (Tukey’s HSD, *p* ≥ 0.05). (EAL = *Enhalus acoroides* leaf; EAR = *Enhalus acoroides* root; HPL = *Halodule pinifolia* leaf; HOL = *Halophila ovalis* leaf; C + = Positive control)

**Fig. 5:**
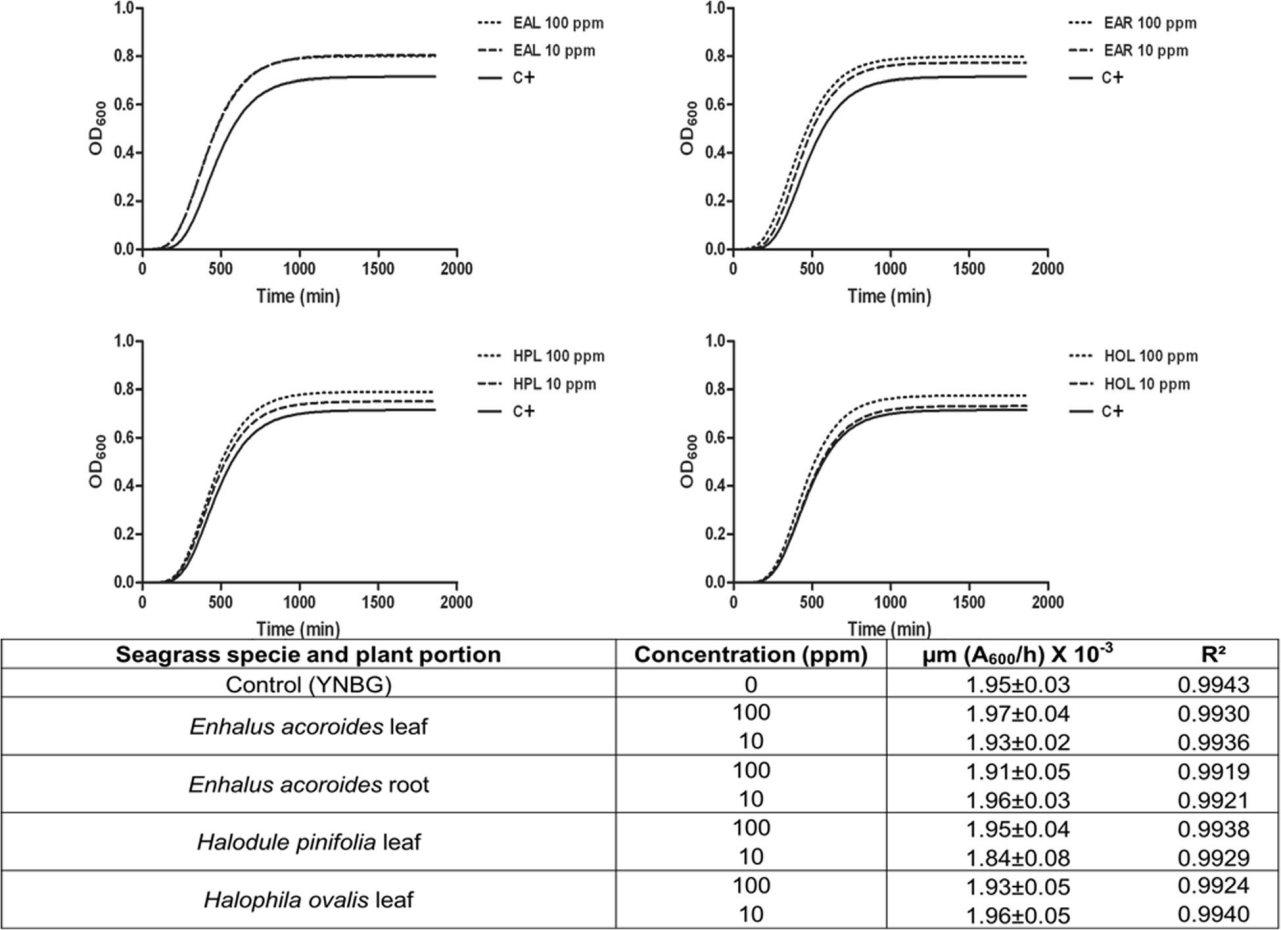
OD-based growth curves of *E. coli* in absence (positive control) and in presence of each seagrass extract at 10 and 100 ppm. Maximum specific growth rate (μ_m_) and the goodness of fit (R^2^) obtained by the Gompertz model. Data represent the mean ± SDs of three independent measurements. Means reported showed no statistically significant differences between the positive control and treated samples (Tukey’s HSD, *p* ≥ 0.05). (EAL = *Enhalus acoroides* leaf; EAR = *Enhalus acoroides* root; HPL = *Halodule pinifolia* leaf; HOL = *Halophila ovalis* leaf; C + = Positive control)

The response of the planktonic growth of the selected microorganisms in the presence of the seagrass extracts at the highest concentrations (10 and 100 mg/l) is reported in Figs. 4 and 5. *C. albicans* and *E. coli* growth rates (table in Figs. 4 and 5) showed that there are no statistically significant differences between the presence and the absence of the extracts obtained from every plant portion at any tested concentration. Therefore, concentrations ≤100 mg/l plant extract were used in the subsequent studies.

### *E. acoroides* leaf extract inhibits cell adhesion on a hydrophobic surface

**Fig. 6:**
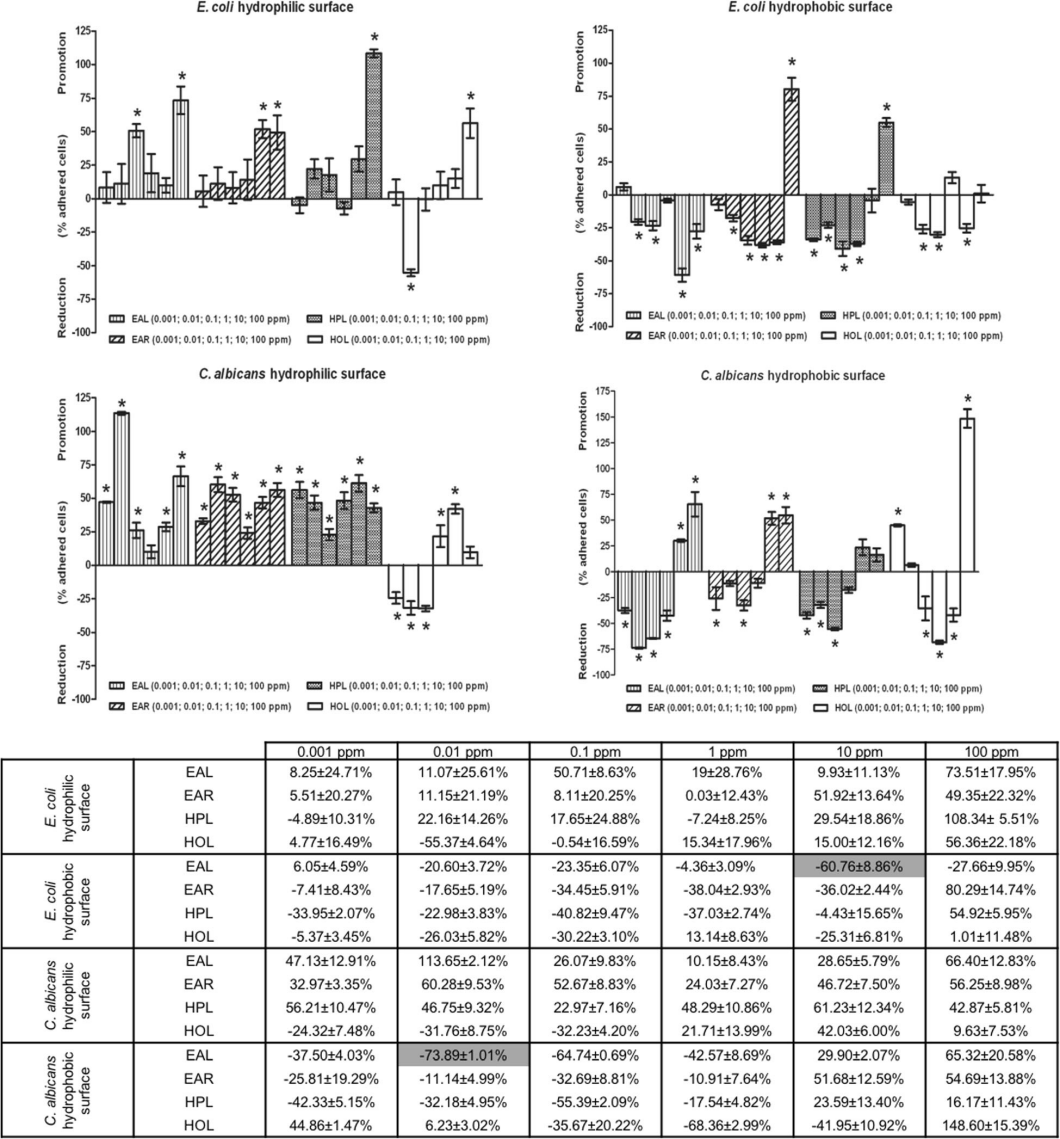
Microplate-based biofilm assay. Percentage reduction of the number of adhered cells of *E. coli* and *C. albicans* on hydrophilic and hydrophobic surface in presence of non-lethal concentrations of seagrass extracts. According to post hoc analysis (Tukey’s HSD, *p* ≤ 0.05), stars indicate statistically significant differences between the means of three independent replicates. In addition, the mean ± SDs of the percentage reduction of the number of adhered cells with seagrass extracts at non-lethal concentrations on hydrophilic and hydrophobic surface are reported in the table. The higher anti-adhesion effect for each microorganism was highlighted. (EAL = *Enhalus acoroides* leaf; EAR = *Enhalus acoroides* root; HPL = *Halodule pinifolia* leaf; HOL = *Halophila ovalis* leaf)

The percentage reduction of the number of adhered cells of *E. coli* and *C. albicans* on hydrophilic and hydrophobic surface in presence of non-lethal concentrations of seagrass extracts is showed in Fig. 6. The results revealed that *E. acoroides* and *H. ovalis* were the most promising extracts for *C. albicans*, with excellent anti-adhesion activity, reducing fungal coverage up to 73.89 ± 1.01% and 68.37 ± 2.49% at 0.01 and 1 mg/l, respectively. For *E. coli*, 10 mg/l of *E. acoroides* leaf extract was found to be the concentration with the highest reduction in cell adhesion (reduction of bacterial coverage by 60.86 ± 8.85%). Therefore, 0.01 mg/l and 10 mg/l *E. acoroides* leaf extract were chosen as the best non-biocidal concentrations for *C. albicans* and *E. coli* respectively, and were used in the subsequent studies.

### *E. acoroides* leaf extract does not impact on biofilm growth curves, but does induce biofilm dispersion in *C. albicans* and interfere with AI2

A CDC reactor was used as the laboratory scale model system to grow a complex and mature *C. albicans* biofilm in the absence and presence of 0.01 mg/l *E. acoroides* leaf extract, the most effective concentration obtained from the adhesion assay.

**Fig. 7:**
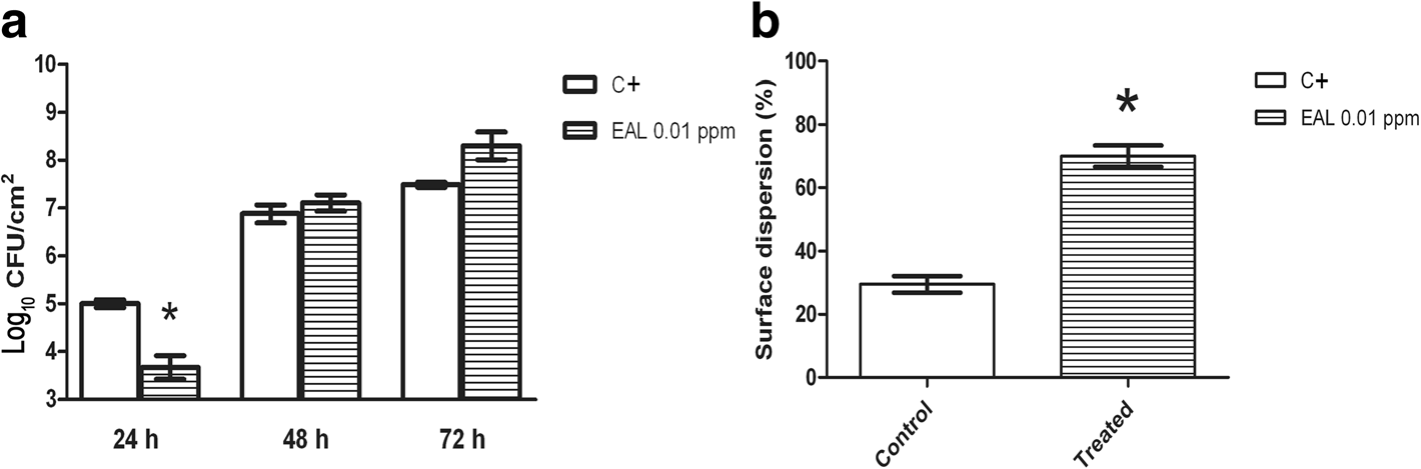
CDC biofilm growth on polycarbonate coupons (**a**) and biofilm dispersion rate (**b**) of *C. albicans* in absence (positive control) and in presence (treated) of 0.01 ppm of *Enhalus acoroides* leaf extract. Stars indicate statistically significant differences (Tukey’s HSD, *p* ≤ 0.05) between the means of three independent replicates. (C + = Positive control; EAL = *Enhalus acoroides* leaf)

Results in Fig. 7a indicated a significant reduction in the number of viable cells adhered on coupon surfaces treated with the extract, compared to the untreated ones, after 24 h (reduction of fungal coverage up to 26.77 ± 9.01%). Coupons collected after 48 and 72 h showed no significant differences between the treated biofilm and the control.

A significant increase in the number of dispersed cells in the treated biofilm (70 ± 6.83%) was observed (Fig. 7b).

**Fig. 8:**
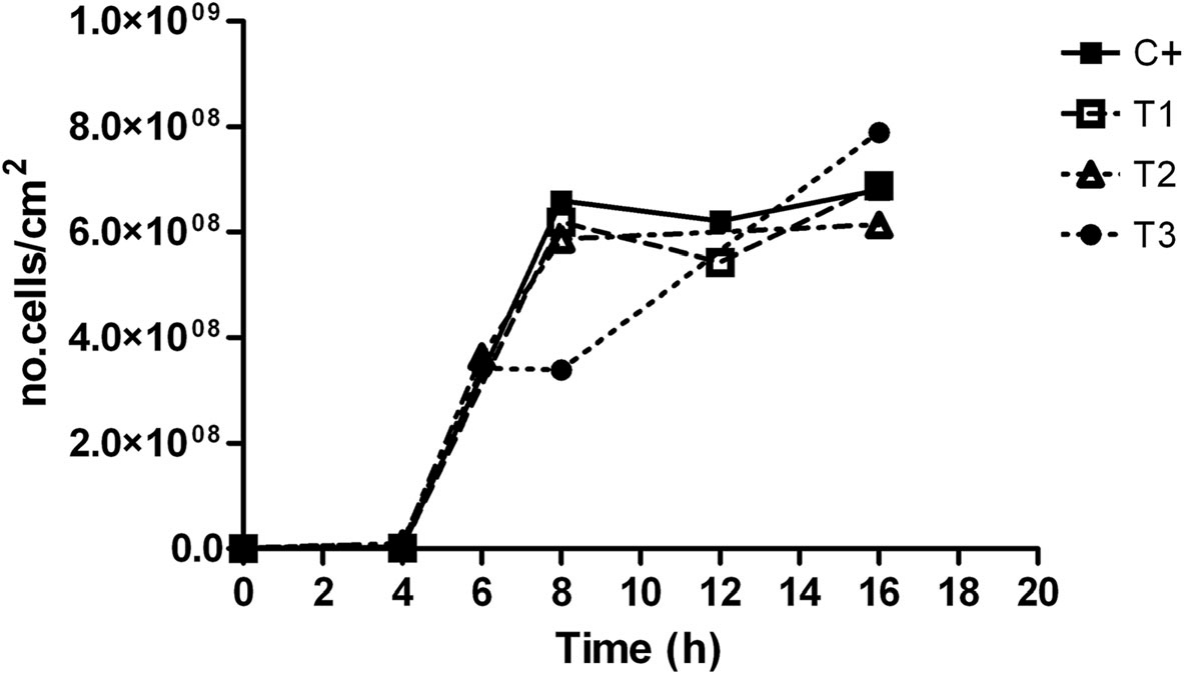
Biofilm growth at the solid/air interface. *E. coli* biofilm grown on polycarbonate membrane under three experimental conditions: i) treatment 1: growth in contact with 1 ml of LB with 10 ppm of *E. acoroides* leaf extract; ii) treatment 2: overnight culture grown with 10 ppm of *E. acoroides* leaf extract and then growth in contact with 1 ml of LB; iii) treatment 3: overnight culture grown with 10 ppm of *E. acoroides* leaf extract and growth in contact with 1 ml of LB with 10 ppm of *E. acoroides* leaf extract. In the positive control, microorganisms grew in 1 ml LB inside a basolateral well without the extract. Data obtained were divided by the area of the membrane, and means were reported. The experiment was repeated three times. (T1 = treatment 1; T2 = treatment 2; T3 = treatment 3; C + = Positive control)

A colony biofilm assay was used to grow a complex and mature *E. coli* biofilm in the presence and absence of 10 mg/l *E. acoroides*. Results in Fig. 8 showed no significant reduction in the number of viable cells during biofilm formation on the membrane treated with the extract, compared to the untreated, after 18 h in all the experimental conditions. Treatment 3 showed a growth rate slowdown in the interval 6–8, in which *E. coli* cells were in contact with the extract during both overnight growth and biofilm formation (reduction of cellular growth, compared to the control, up to 48.64 ± 4.02%). This growth curve was characterized by two exponential phases separated distinctly by an intermediate phase where the growth rate is very low. After that, at 16 h the number of viable cells was similar to the other treatments.

**Fig. 9:**
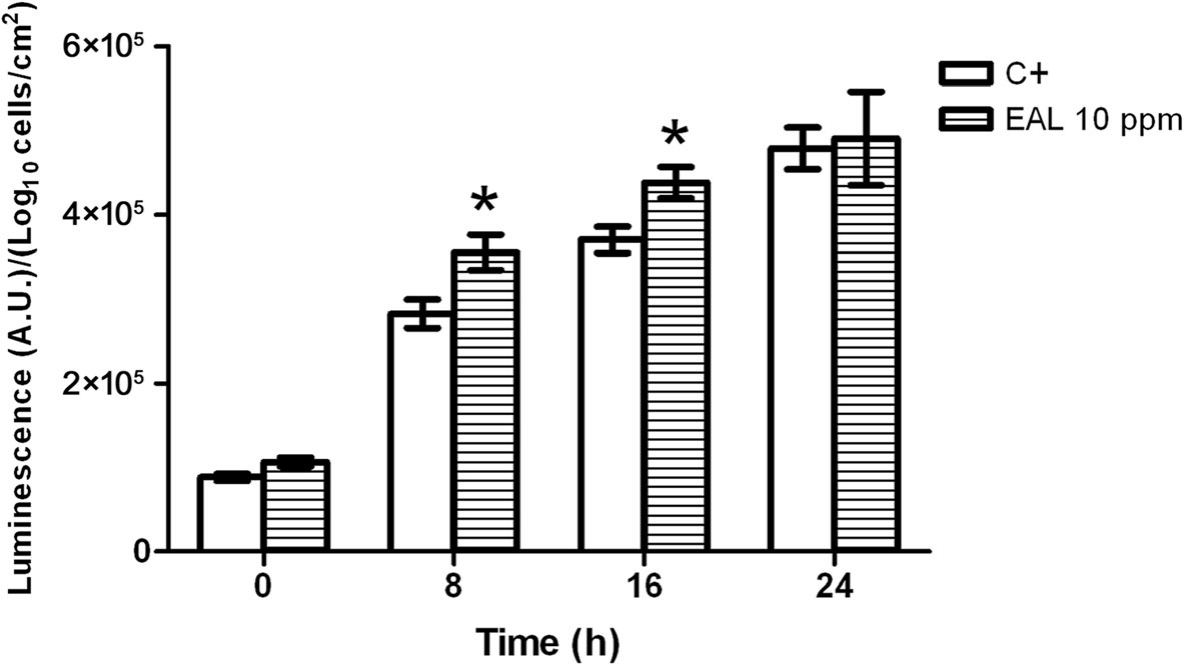
Relative luminescence emitted by *Vibrio harveyi* in absence (positive control) and in presence of 10 ppm of *E. acoroides* leaf extract for 24 h. The relative luminescence has been calculated by normalizing luminescence by the number of adhered cells. Stars indicate statistically significant differences (Tukey’s HSD, p ≤ 0.05) between the means of three independent replicates. (C + = Positive control; EAL = *Enhalus acoroides* leaf)

The effects of 10 mg/l of *E. acoroides* leaf extract on the cellular communication of *V. harveyi* were reported in Fig. 9. The results highlighted a significant increase in the relative luminescence emitted at time 8 h compared to the control (25.75 ± 7.49%).

## Discussion

Biofilm resistance to antimicrobial agents is a major worldwide health care issue. Therefore, a successful reduction of surface colonization can be a potential strategy for the management of unwanted biofilms, especially on medical devices and work surfaces.

In this context, the use of plant-derived extracts to modulate biofilm genesis and dispersion may be a viable alternative. The present study is the first report describing the antibiofilm efficacy of non-lethal concentrations of *E. acoroides*, *H. pinifolia* and *H. ovalis* methanol extracts in counteracting microbial biofilms, highlighting the possibility that the selected seagrass species act as an extracellular signal mediating their biofilm activities.

*E. coli* and *C. albicans* were chosen as model systems for bacterial and fungal infections, respectively. *E. coli* biofilms are found to be the major causative agent of many intestinal infections, for recurrent urinary tract infections, and it also responsible for indwelling medical device-related infectivity [43]. *C. albicans* is one of the very few fungal species causing disease in humans. These infections range from superficial mucosal and dermal infections, such as thrush, vaginal yeast infections, and diaper rash, to vascular catheters and dental implants infections [44].

The bioactive properties of the seagrass species selected in this work are well known, and have been reported in detail by several authors [45–47]. However, until now attention has mainly focused on the antimicrobial activity of seagrass extracts, which, through disk diffusion assays, were investigated not in their capacity as biofilm-forming microorganisms but in their planktonic state. Using lethal concentrations, Umamaheshwari et al. [46] reported the antibacterial activity of *H. ovalis* and *H. pinifolia* extracts, obtained using different solvents, against different microbial strains, recording maximum antibacterial activity by the ethanol extract of *H. pinifolia*. Instead, Choi et al. [48] reported the antimicrobial properties of *Zostera marina* methanol extract and its organic solvent fractions on three human skin pathogens (*Staphylococcus aureus*, *S. epidermidis* and *C. albicans*), and Natrah et al. [47] reported the antibacterial properties of methanol extracts of *E. acoroides* and other seagrass and seaweed species on different aquaculture pathogens (*Aeromonas hydrophila*, *Vibrio alginolyticus*, *V. parahaemolyticus*, *V. anguillarum* and others).

In contrast, to the best of our knowledge, no papers have investigated the antibiofilm activity of *Enhalus acoroides, Halodule pinifolia* and *Halophila ovalis* at non-lethal concentrations against bacterial (*E. coli*) and fungal (*C. albicans*) biofilms. To this end, methanol extracts, obtained from different organs of three seagrass species (namely, *Enhalus acoroides* leaves and roots, *Halophila ovalis* leaves and *Halodule pinifolia* leaves) were screened for their ability to modulate biofilm genesis without killing cells. Methanol was used as the extraction solvent, having been previously reported as the most effective solvent to obtain high concentrations of bioactive compounds with antibacterial activity from seagrasses, compared to other extraction solvents [45, 49, 50].

Before evaluating the antibiofilm activity, the extracts, at concentrations of 100 mg/l, were first proved to not act as a carbon and energy source nor to affect the cellular growth of *C. albicans* and *E. coli*. Therefore, concentrations ≤100 mg/l plant extract were used in the subsequent studies.

With the aim of investigating the effects of seagrass extracts on cell adhesion to surfaces, the first step of biofilm formation, microtiter based assays were performed. The results revealed excellent anti-adhesion activity for *E. acoroides* leaf extract, reducing fungal coverage up to 74% and bacterial coverage up to 61% at 0.01 and 10 mg/l, respectively. Therefore, 0.01 mg/l and 10 mg/l *E. acoroides* leaf extract were chosen as the best non-biocidal concentrations for *C. albicans* and *E. coli* respectively, and were used in the subsequent studies. These concentrations significantly decreased the number of adhered cells on a hydrophobic surface, more so than on the hydrophilic one. Previous studies had highlighted the preference for hydrophobic surfaces, these reporting a decreased adhesion on the hydrophobic surface compared to the hydrophilic [51, 52]. This is probably due to the hydrophobic nature of the aerial surfaces of plants [53].

In the present study the anti-adhesion activity of the seagrass extracts was dose-dependent, but the highest concentrations did not correspond to those with the best performance. Indeed, several studies have reported a weak activity of the compounds at low and high concentrations, and excellent activity at intermediate concentrations [54]. Such a response, widely known in literature, is defined as hormesis, an adaptive behavior of microorganisms to provide resistance to environmental stress and improve the allocation of resources to ensure cell stability [19, 55].

To further explore the effect of the most promising seagrass extract on biofilm development and detachment, CDC reactors were employed to reproduce biofilm at the solid/liquid interface, while for the assessment of the antibiofilm effect in the adhesion phase microplate-based biofilm assays are the most suitable [41, 56, 57]. In this study, a significant reduction in fungal coverage (up to 26.77 ± 9.01%) after 24 h (static adhesion phase) was observed in presence of 0.01 mg/l *E. acoroides* leaf extract. This result confirms the anti-adhesion activity observed in microtiter assays. Coupons collected after 48 and 72 h showed no significant differences between treated and control samples.

In order to assess the possibility of 0.01 mg/l *E. acoroides* leaf extract to promote *C. albicans* biofilm-detachment from the surface of coupons, a biofilm dispersion assay was performed. Results showed a significant increase in the number of dispersed cells in the treated biofilm, compared with the untreated (70 ± 6.83%), suggesting a further mechanism of action for the seagrass extract as biofilm dispersing agent. In fact, the phase of biofilm dispersion could be an interesting target for the development of new antibiofilm strategies, forcing the planktonic state and reestablishing the efficacy of traditional antimicrobial agents [4, 58]. Literature with information related to *C. albicans* biofilm dispersion is scarce. Farnesol and cis-2-decenoic acid showed dispersion-promotion of microcolonies of *C. albicans* biofilm [58, 59]. In addition, Villa et al. [60] reported that non-lethal concentrations of *Muscari comosum* ethanol bulb extract can modulate yeast adhesion and subsequent biofilm development on abiotic surfaces, and such concentrations could provide an extracellular signal responsible for biofilm dispersion.

For *E. coli*, the CDC reactor was not suitable to evaluate the possible effects of the extracts on the biofilm stages*.* Also other authors have reported the poor biofilm formation exhibited by *E. coli* K-12 strain under hydrodynamic conditions [61–63]. The effect of 10 mg/l of *E. acoroides* leaf extract on *E. coli* biofilm formation was then evaluated using a membrane-supporting biofilm reactor, which allowed the formation of a biofilm at the solid/air interface. This technique forced the cells to attach to a surface, a feature that allowed direct investigation of the effect of the selected extract on the development of the biofilm, whilst bypassing the effect on the adhesion phase.

No significant reduction in the number of viable cells during biofilm formation on the membrane treated with the extract, compared to the untreated, after 18 h in all the experimental conditions was observed. Treatment 3 showed a growth rate slowdown in the interval 6–8 h, in which *E. coli* cells were in contact with the extract during both overnight growth and biofilm formation (reduction of cellular growth, compared to the control, up to 48.64 ± 4.02%). Interestingly, treatment 3 showed a biphasic growth curve compared with the growth curves of the other treatments, a trend that could be explained by the bioluminescence produced by *V. harvey*. As signaling molecules play an important role in biofilm development and detachment, the effects of 10 mg/l of *E. acoroides* leaf extract were investigated using *V. harveyi*, suggesting other possible antibiofilm mechanisms of action of compounds in the chosen seagrass extract. The results revealed that at time 8 h, the samples treated with the leaf extract showed a significant increase in the relative luminescence emitted, compared to the control (25.75 ± 7.49). Villa et al. [64] reported an increase of autoinducer-2 (AI-2) activity and a reduction in biofilm formation in *E. coli* cells treated with zosteric acid, a phenolic compound occurring in the seagrass *Zostera marina*. In fact, it has been hypothesized that the accumulation of AI-2 above a threshold level leads to reduced biofilm formation due to the induction of a hypermotile phenotype that is unable to adhere to the surface [64]. Huber et al. [65] demonstrated that some polyphenolic compounds containing a gallic acid residue commonly produced by some plant species inhibited intercellular communication in bacteria. Truchado et al. [66] reported the ability of some phytochemical compounds (cinnamaldehyde, ellagic acid, resveratrol, rutin and pomegranate extract) to interfere with the quorum sensing system of *Yersinia enterocolitica* and *Erwinia cartovora*.

It has been well known that the antibiofilm activity of plant extracts is closely linked with the content of secondary metabolites, such as phenols and/or flavonoids, which represent the total amount of phenolic compounds in a plant extract [13]. The phenolic compound content is also deeply associated with the antioxidant activity of plant extracts [67]. Therefore, we determined the total phenolic acid (TPC) and flavonoid (TFC) content and the antioxidant activity (ORAC) of methanolic extracts in order to highlight features of the most promising antibiofilm extract, the *E. acaroides* leaf extract. Results show that *E. acaroides* leaf extract presents the lower TPC and TFC values compared to other seagrasse extracts. Although the low content of phenolic compounds, the *E. acaroides* leaf extract displays a higher ORAC value compared to the root extract. This indicates the abundance of other, non-phenolic compounds with antioxidant capacity in the leaves of *E. acoroides*. Cattò et al. [39] suggested the importance of antioxidant compounds in hindering biofilm formation. The researcher discovered that the mechanism of action behind the antibiofilm performance of zosteric acid, a secondary metabolite of the seagrass *Zostera marina*, is related to the antioxidant activity of the molecule, and its interaction with the WrbA protein responsible maintaining cellular homeostasis and defense against oxidative stress.

To gain more insight into possible antibiofilm compounds in the seagrass extracts, individual substances in the methanolic extract were analyzed by LC-MS. Preliminary analysis shows that the phytochemical profile of the *E. acaroides* leaf extract is mainly characterized by the presence of the flavones apigenin and luteolin, three kaempferol derivates and the carboxylic acids benzoic and azelaic acid. This unique quantitative and qualitative chemical composition confers antibiofilm properties to the *E. acaroides* leaf extract.

Some of these compounds have shown to exhibit antibiofilm properties at non-lethal concentrations. Kaempferol, apigenin and luteolin from red wine reduced biofilm formation of methicillin-sensitive *S. aureus* significantly [68]. Sánchez et colleagues [69] reported that sub-lethal concentrations of plant extracts inhibit *E. coli* and *S. aureus* biofilms. The antibiofilm properties of the extracts were associated to the presence of flavonoids, such as kaempferol and apigenin, which modulate bacterial cell-cell communication by suppressing the activity of the autoinducer-2 [70]. However, we should keep in mind that the antibiofilm effects of plant extracts could be the result of interactions among different components of the extract at specific concentrations, and not only due to the effects of a single, predominant compound [4, 71].

## Conclusions

In conclusion, the *E. acoroides* leaf extract proved to be the most promising extract among those tested. Indeed, the selected non-lethal concentrations of *E. acoroides* leaf extract were found to exert an antibiofilm effect on *C. albicans* and *E. coli* biofilm in the first phase of biofilm genesis, opening up the possibility of developing preventive strategies to hinder the adhesion of microbial cells to surfaces. The leaf extract also affected the dispersion and maturation steps in *C. albicans* and *E. coli* respectively, suggesting an important role in cell signaling processes. These effects could be explained by the presence of active compounds like kaempferol and apigenin at specific concentrations in the extracts of *E. acoroides*, which are known to possess biofilm inhibiting properties. Furthermore, there could be a synergistic action of these flavonoids with other compounds occurring in the plant, enhancing the global antibiofilm effect. Currently, the leaf extract is being investigated with the objective of testing fractions for identifying the active compounds and to better understand the mechanisms of action of this seagrass species.
